# “Nonperturbative Nonlinearities”: Perhaps Less than Meets the Eye

**DOI:** 10.1021/acsphotonics.4c00645

**Published:** 2024-08-07

**Authors:** Jacob B. Khurgin, Nathaniel Kinsey

**Affiliations:** † Department of Electrical & Computer Engineering, Johns Hopkins University, Baltimore, Maryland 21218, United States; ‡ Department of Electrical & Computer Engineering, Virginia Commonwealth University, Richmond, Virginia 23284, United States

**Keywords:** Nonlinear Optics, Epsilon-Near-Zero, Transparent Conducting Oxides, Nonperturbative Nonlinearities, Optical Materials

## Abstract

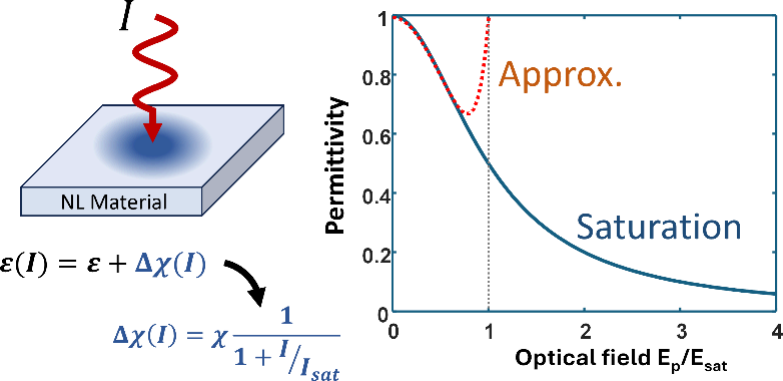

We address challenges in characterizing changes in permittivity and refractive index beyond standard perturbative methods with special attention given to transparent conductive oxides (TCOs). We unveil a realistic limit to permittivity changes under high optical power densities. Our study covers both slow and ultrafast nonlinearities, demonstrating that all nonlinearities induce refractive index changes accurately described by a simple curve with saturation electric field (or irradiance) and maximum change of permittivity at saturation. Our model, grounded in material properties, like oscillator strength and characteristic times, offers a robust framework for understanding and predicting nonlinear optical phenomena in TCOs and other materials. We differentiate between the significance of higher-order nonlinear susceptibilities in ultrafast and slow nonlinear scenarios. We aim to provide valuable insights for researchers exploring strong light–matter interaction.

## Introduction

Recent developments in nonlinear optics have been focused on applications where very large and fast changes of permittivity (refractive index) are required, such as adiabatic frequency conversion,^1,2^ time reflection and refraction,^3^ time crystals,^4,5^ and so on. With that demand, a variety of new (or previously overlooked) material systems are being explored, including intersubband transitions in quantum wells for the mid-IR region,^6^ plasmonic metals,^7,8^ and most prominently, transparent conductive oxides (TCOs) usually operating in the “epsilon-near-zero” (ENZ) regime for the near-infrared (NIR) region.^9−13^ In these instances, significant emphasis has been placed on the observation that, at moderate-to-high pump intensities (10–100 GW/cm^2^), alterations in permittivity defy straightforward descriptions using conventional chains of nonlinear susceptibilities. This is especially true for the case of TCOs which are indeed showing impressive results of index change on the scale of tens to one hundred percent.^14−16^ In response, the result is lauded as a new “nonperturbative” realm in nonlinear optics.

As the discussion around nonperturbative nonlinearities begins to rise, it is worthwhile to pause and examine two key questions surrounding these effects and their descriptions:

(1) Does exhibiting a nonperturbative response represent something unconventional, and can it lead to previously unknown phenomena?
(2) What insights do we gain in physical understanding through the use of nonlinear susceptibility formalism?

The first question has a straightforward answer. For any response, under small excitation, one can introduce a perturbative response as is commonly done. However, at some point the phenomena will tend to deviate or saturate—a well-documented case for basic elements such as mass-spring oscillators, Ohm’s law, and transistor gain just to name a few. Close to saturation, the response can no longer be expanded in a power series and the typical perturbative approximation is no longer valid. In optics, this effect can be readily seen for modifications in the permittivity ε(ω) of transparent materials (we knowingly stay away from the nontransparent materials such as metals since large loss in them impedes their practical applications). In this case, the lower bound of permittivity for fully transparent materials is 0 while the upper bound on the refractive indices for all known materials is approximately 4 in the visible and NIR regions^17^ (slightly higher in the mid-IR). Therefore, there exists an upper limit for permittivity modulation. Depending on the sign of nonlinearity, it must always saturate either at the lower or upper bound of permittivity. In each case, we demonstrate that the change in permittivity can be analytically described with relative ease, and no unexpected phenomena emerge. This holds true for any type of nonlinearity and for any transparent material, as demonstrated in the remainder of this article.

The second question presents a greater challenge to answer. Upon exploring various nonlinearities, we conclude that while nonlinear susceptibilities are indispensable in describing all types of parametric processes such as harmonic, sum, and difference frequency generation, in most other instances, susceptibilities do not offer additional insights into the underlying physics of the nonlinearity. In fact, they can be deceptive. In general, we show that the use of nonlinear susceptibilities can make sense for “fast” processes, where the effects are associated with nonlinear polarizabilities (hyperpolarizabilities) of various electronic states. However, their use is not justified for processes, where the polarizability change is not directly imposed by the optical field but is caused by an indirect action such as a rise in electronic temperature. In these cases, the use of the nonlinear index and its dependence on pump power is more than sufficient to describe the physics behind the nonlinearity.

To highlight these points and explore the realm of nonperturbative nonlinearities, we analyze nonlinear index change, i.e. odd-order processes, manifesting themselves in such phenomena as self- and cross-modulation as well as four-wave (and higher) mixing. We begin our discourse with a discussion of “slow” processes in a classic two-level system to introduce the saturable nonlinear index model while developing connections between the regions where a perturbative expansion is and is not valid. Next, we switch our focus to the case of the ENZ region of TCOs, outlining terminology of nonperturbative effects and developing a saturable nonlinear index description for intraband optical excitations. Lastly, we expand the discussion to the case of “fast” nonlinearities arising from virtual population oscillations and illustrate that, even in this case, a saturation model is sufficient to describe the resulting intensity dependence.

Before delving into distinct cases of nonlinearity, it is necessary to make two general statements. First, we are exclusively focused on the intrinsic nonlinearity of the material’s dielectric permittivity versus the magnitude of the electric field inside the material. It is well-known that employing various resonant schemes (such as microcavities, metasurfaces, nanoantennas, various “slow light” schemes, etc.) leads to the enhancement of the field inside the material, as well as an increase in effective interaction time between light and matter. This enhancement can boost the “effective” nonlinearity, evidenced by sharp changes in phase delay and/or transmission, and can even result in clearly “nonperturbative” switching via optical bistability. These resonant enhancement and switching schemes have been well documented and understood over many years, but what ultimately drives them is still the intrinsic nonlinearity of the material, which is our primary focus.

Second, while we concentrate on permittivity dependence, for the sake of comparison with the literature, we sometimes introduce nonlinear index change. However, it should be stressed that this index dependence is solely related to the irradiance inside the bulk nonlinear material; thus, it is not influenced by any resonant or slow light enhancement.

## Nonperturbative Regime in Slow (Real) Nonlinearities

Slow^18^ nonlinearities involve the absorption of pump light, leading to various physical processes such as absorption saturation,^19−21^ heating,^22^ or carrier diffusion.^23^ These processes result in a change in the permittivity at the probe wavelength. In cases where the pump and probe are distinct, phenomena such as cross-phase modulation and four-wave mixing are observed. If the pump and probe are identical, the phenomenon known as self-phase modulation occurs. From the outset, it is crucial to clarify that slow in this context refers to the modification of real excitations with a specific characteristic time, not an absolute measure of speed. The characteristic time can vary widely among processes, making even slow optical phenomena as fast as a few femtoseconds.

**Figure 1:**
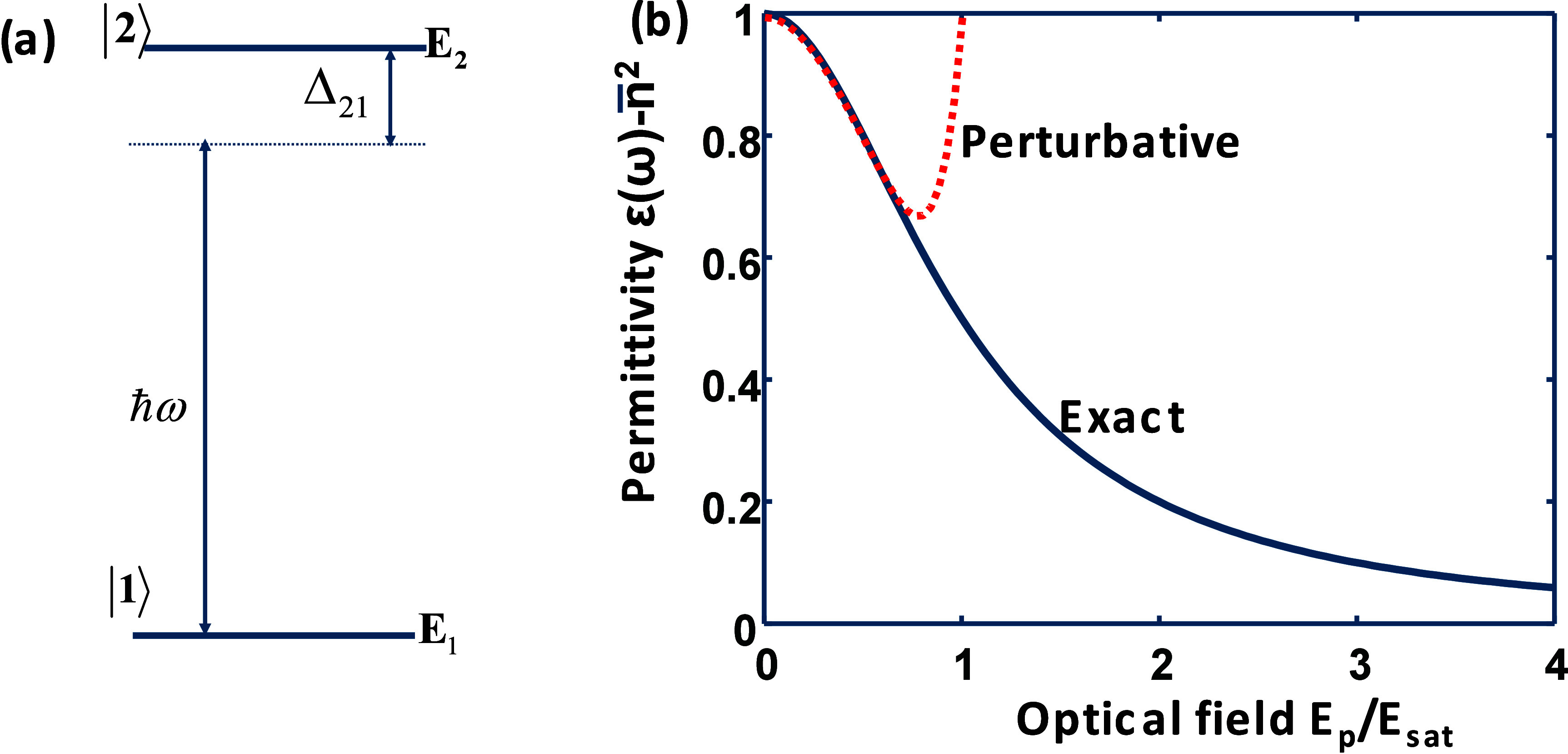
(a) Two-level system not far from resonance. (b) Susceptibility of the two-level system χ_2level_(ω)=ε(ω) – *n̅*^2^ as a function of the pump field. Solid line, exact; dashed line, perturbative approximation up to χ^(7)^.

To ascertain the importance of nonperturbative effects for slow processes, we start with the simplest textbook example–the well-known case of saturable absorption in a two-level system. Consider, for example, a two-level system as shown in Figure 1a. This system is characterized by the transition energy dipole matrix element **d**_12_=⟨1|*e***r**|2⟩, relaxation time τ_21_, and broadening Γ (note that the subsequent analysis can be extended to situations where two levels represent energy bands in a solid by integrating over the density of states in energy space). At the frequency ω, the permittivity can be found as
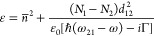
1where *n̅*^2^ is the permittivity due to all the transitions except the one between levels 1 and 2, and *N*_1_,*N*_2_ are the level populations. Here, we consider the *n̅*^2^ term as constant but note that these transitions have their own nonlinearities that may play a role in strong pumping regimes or in cases where additional frequencies are generated near their resonance.

Building from eq 1, we can introduce the absorption cross-section as
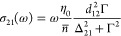
2where the detuning is defined as . Now, if we introduce a strong pump with power density *I*_P_ at frequency ω_p_, the upper level becomes populated, and the population difference saturates as
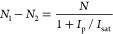
3where *N* is the density of active entities (atoms, molecules, ions, etc.) and *I*_sat_ is the saturation power density given by
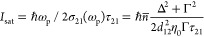
4

The permittivity (real part) at the probe frequency ω is then:
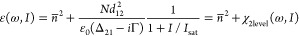
5where χ_2level_(ω,*I*) is intensity-dependent susceptibility of the two-level system which does not include susceptibility due to nonresonant states. The refractive index is then given by
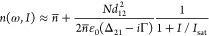
6

The real part of susceptibility χ_2level_(ω,*E*_p_) relative to its unsaturated value χ_2level_(ω,0) is plotted in solid blue in Figure 1b versus the optical field of the pump relative to the saturation field .

This expression is quite sufficient to describe the nonlinearity almost exactly and is necessitated by the saturation of the population difference which, as can be easily seen from eq 3, must be bounded between 0 and *N*.

Furthermore, to describe phenomena such as four-wave mixing one can use the square of the sum of pump *E*_p_ and probe *E*_pr_ fields, (*E*_p_ + *E*_pr_)^2^*n*/2η_0_, in place of *I*_p_ and use eq 5 and eq 6 for as long as the beat frequency between pump and probe is much less than 1/τ_21_.

While the prior case did not require a perturbative assumption, we can relate it to this case by introducing the nonlinear susceptibility through a power series expansion of eq 5 to obtain
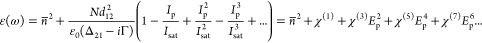
7where the pump field is . We can isolate the odd-order nonlinear susceptibilities as
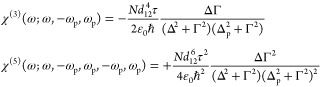
8and so on, where we have introduced the detuning values Δ_p_ for the pump and Δ for the probe. A similar result could be obtained using perturbation theory, starting from the equation for the density matrix and evaluating the off-diagonal density matrix element, i.e., polarization as a series ρ_21_(ω) = ρ_21_^(1)^+ρ_21_^(3)^+ρ_21_^(5)^+···

As shown in Figure 1b, the change in permittivity using this approximation for terms up to χ^(7)^ is shown as the orange dashed line. As expected, the expansion provides a good approximation for small applied fields but deviates as one approaches the saturation field. We can then delineate between the perturbative and nonperturbative regimes by noting that *I*_sat_ is exactly the point when the two curves in Figure 1b fully diverge.

Given the limited range of validity, the question is what benefit does this description provide? By observing eq 8, we can see that the perturbative approach, with nonlinear susceptibilities, abstracts material parameters that may not be readily available (e.g., *d*_12_), combining them inside coefficients that can be extracted from experiments and easily displayed in databases or textbooks. While useful, this description loses the dependency on material parameters such as relaxation rate, detuning, etc., and as a result, coefficients can vary drastically based on the material growth conditions (e.g., bandgap, scattering rate) and excitation parameters (e.g., detuning, pulse width). Thus, the generality of susceptibility values is significantly reduced, giving rise to a wide range of values for slow nonlinear transitions in materials such as semiconductors and metals.^24^

Whether the reduced generality is a suitable price to pay depends upon the application. However, what is perhaps the most egregious issue with the use of susceptibilities to describe slow nonlinearities is the loss of the ability to quantify the validity range which can lead to an all-too-common extrapolation of the nonlinear response or the reporting of coefficients at or beyond the saturation regime.

In this sense, it is important to remember that nonlinear susceptibilities are usually introduced as the measure of the polarizability of electronic states, and the nonperturbative regime is achieved when nonlinear polarization becomes comparable to linear polarization. For fast or virtual nonlinearities, this occurs when the pump field approaches the “atomic field” of the state in the atom, molecule, or bond within a solid. The scale of that field is , i.e., a few volts per angstrom. But for slow or real nonlinearities, one enters the “nonperturbative” regime when *I*_p_ ∼ *I*_sat_ described by
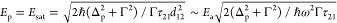
9

In this case, we stress that the onset of the nonperturbative regime has little to do with the atomic field and everything to do with operation near resonance Δ_p_, line width Γ, and most critically, the relaxation time τ_21_, which can vary by many orders of magnitude–from milliseconds to femtoseconds (as in TCO’s discussed in the next section). Ultimately, near resonance one obtains the onset of the nonperturbative regime as
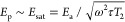
10where is a coherence time. The characteristic time in eq 10 is τ = τ_21_ except for short pulse durations where τ_p_ < τ_21_, where we take τ = τ_p_. A similar analysis can be performed for other slow nonlinearities such as thermal,^22^ photorefractive,^23^ etc. with the only difference being the characteristic time τ. As a result, it is possible to reach saturation at relatively low pump power densities, which can invalidate a perturbative expansion well before expected. As an example, for a free carrier generation process at 1 μm with *T*_2_ = 10 fs and τ = τ_p_= 1 ps, *E*_sat_ = ∼(1/200)*E*_a_.

The main conclusion is that at high powers when *I*_p_ exceeds *I*_sat_, the expansion in eq 7 is no longer valid. However, this does not present any difficulty, as the exact solution in eq 5 is readily available. Moreover, it should not be considered as anything unusual. In this sense, introducing nonlinear susceptibilities for slow nonlinearities is not necessary and it does not reveal any underlying physics, in fact, it obscures them in favor of simplicity. It is always preferable in our view to use the complete and exact expression for the change of permittivity or index when working with slow nonlinearities. However, for relatively low values of intensity, *I*_p_ ≪ *I*_sat_, we note that an effective nonlinear refractive index is a perfect way to describe the slow nonlinearity. By linearizing eq 6 we can define:
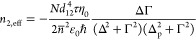
11

By combining this with eq 6, we can obtain the expression for the intensity dependence of the nonlinear index that is valid at any pump power:
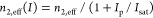
12

We would like to point out that for many popular highly transparent amorphous or crystalline materials (e.g., silica, sapphire, etc.), the perturbative case is not a bad assumption. This is reasonable because nonlinear effects arise from fast or virtual processes that are indeed well described by susceptibilities, and the saturation field of such materials is extremely high, such that other limiting factors take precedence (e.g., ablation, chemical change). But even then, as shown in “A Very Special Case: Nonperturbative Regime in Transparent Conductive Oxides,” one can obtain exact expressions without reverting to the perturbative picture.

Thus, we see that the definition of a nonlinear index coefficient along with a saturation intensity, as outlined in eq 12is the ideal way to quantify the refractive nonlinearities in materials that exhibit a slow nonlinearity.

Having said that, we would like to digress a bit and consider one situation in which the introduction of nonlinear susceptibilities may shed some light on the physics underlying an important and practical phenomenon, stimulated emission depletion (STED) microscopy.^25,26^ Depletion of stimulated emission (or gain) follows the same dependence as the saturation of permittivity [e.g., 1/(1 + *I*/*I*_sat_)]. When a sample is illuminated by a donut-like depletion beam *I*_d_, the gain remains unsaturated only in a small region near the center. This region gets smaller with increasing *I*_d_, and is thus able to exceed the diffraction limit. If one expands the expression for gain into a power series, one can obtain a series of multiphoton processes, χ^(3)^ is a two-photon process, χ^(5)^ is a three-photon process, and so on. Since n-photon imaging has a resolution that is n-times better than a 1-photon process,^27^ it readily explains why STED imaging has superior resolution.^26^

## A Very Special Case: Nonperturbative Regime in Transparent Conductive Oxides

Let us turn our attention to one of the more recent topics in this discussion: TCOs in the ENZ regime. These materials, including compounds such as In:Sn_2_O_3_ and Al:ZnO, have reignited this discussion due to their ability to demonstrate a change in the refractive index on the scale of Δ*n* = ∼0.1–1 in a spectral region where the unmodulated index is on the scale of *n* = ∼0.2–0.5.^10,28^ This has been achieved through the unique combination of slow light enhancement within the ENZ region, the use of slow free carrier nonlinearities, and high damage thresholds in the films.^18,29^ As a result, achieving a 100% modulation of the refractive index is readily feasible, giving rise to discussions surrounding nonperturbative effects.

In this context, it is important to first pause and note a subtle difference in the use of the phrase nonperturbative. As previously outlined, nonperturbative effects arise from a situation in which one employs extreme irradiances and fields commensurate to the interatomic field. In this case, one can consider that the traditional perturbative power series expansion of the atomic polarization breaks down, a case often termed nonperturbative. However, nonperturbative has also been used to describe the following simplification en route to defining the nonlinear refractive index as in eq 7:
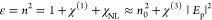
13wherein higher-order susceptibilities are neglected. For typical materials this is a justified simplification, barring the limitations outlined in the remainder of this commentary, namely because χ^(1)^ ≫ χ^(3)^|*E*(ω)|^2^. For example, in the infrared, fused silica has ε ≈ 2.1 while χ^(3)^ ≈ 2.5 × 10^–22^ [m^2^/V^2^], such that typical irradiances of 100 [GW/cm^2^] would generate Δε ≈ 1 × 10^–4^. However, in the case of ENZ materials, the permittivity is near zero to begin with and enhancements to the nonlinear response enable χ_eff_^(3)^ = ∼1 × 10^–17^ [m^2^/V^2^] such that 100 [GW/cm^2^] enables Δε ≈ 0.5. In this case, the optical response is almost entirely defined by the nonlinear permittivity such that the expression in eq 13 is in fact more closely approximated by *n*^2^ ≈ χ^(3)^|*E*_p_|^2^, even for fields that are quite substantially below the atomic field. Now we arrive at an interesting situation in which the permittivity *n*^2^ does increase linearly with intensity, but the refractive index does not. We will term this ‘numerically nonperturbative’ which stands in contrast to the ‘physically nonperturbative’ response outlined in the previous section.

This distinction is important because a hallmark of the ‘physically nonperturbative’ response is the nonlinearity of index change versus applied irradiance which invalidates the use of a singular nonlinear index coefficient. In ENZ materials, one can be ‘numerically nonperturbative’ while not being ‘physically nonperturbative’. This occurs due to the drastic reduction in the steady-state permittivity of the material at the ENZ condition. Simply put, for the expression in eq 13, if *n*_*o*_^2^ = ε ≈ 0 such that *n*^2^ = χ^(3)^|*E*(ω)|^2^ and one directly recovers the ‘numerically nonperturbative’ case even for quite small values of Δε. This is true any material with small permittivity, not just TCOs.

To explore the connection with nonperturbative effects in TCOs at ENZ, we will consider the intraband nonlinear component where both pump and probe reside in the telecommunication region of the spectrum (note that interband nonlinearities are quite similar to the slow nonlinearity outlined in the previous section). While of course not an ideal two-level system, we will see that TCOs in the ENZ regime exhibit a saturation in the modulation of the average effective mass resulting in an intensity dependence that is functionally similar to the two-level system.

**Figure 2:**
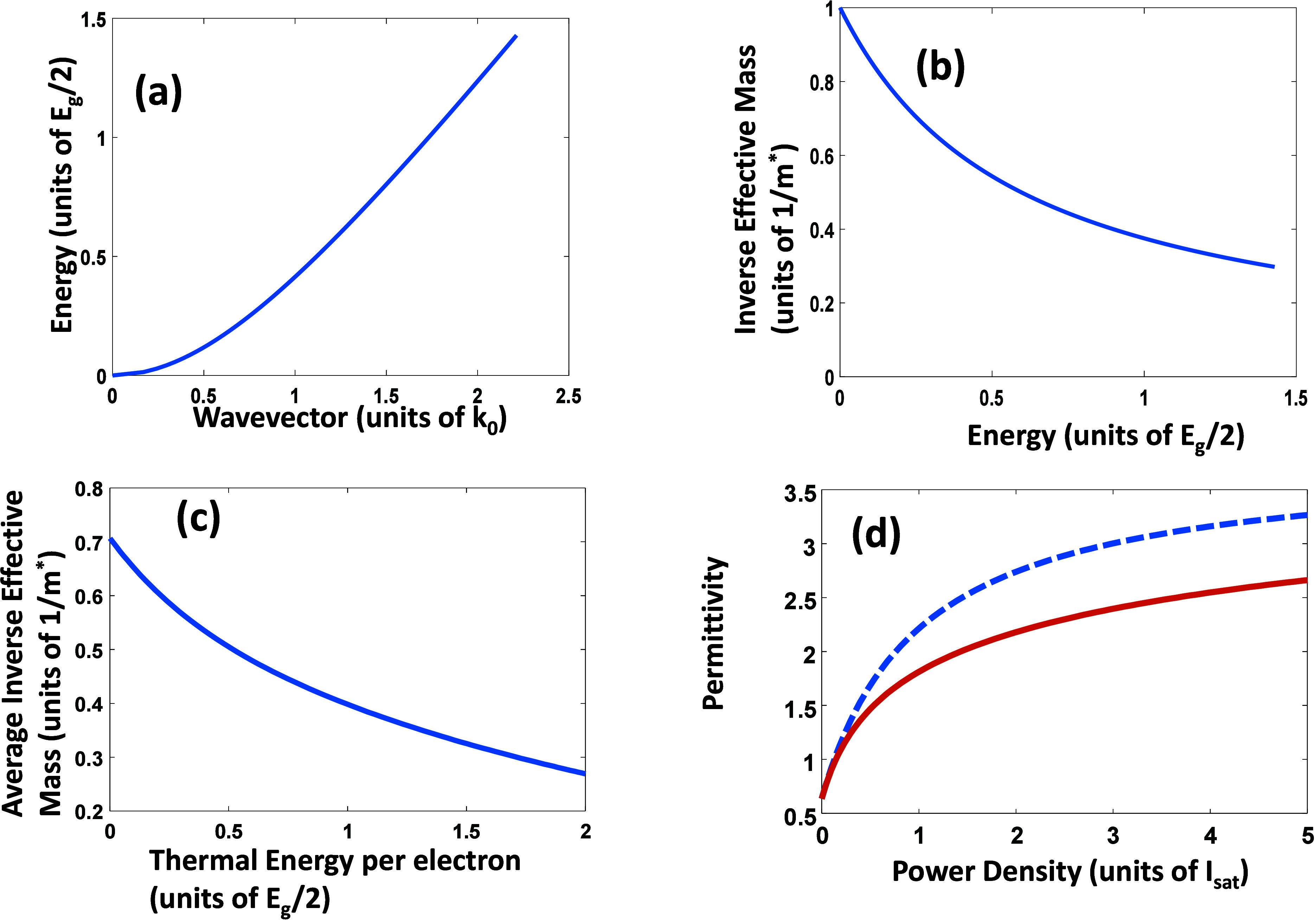
(a) Dispersion *E*(*k*) of nonparabolic band. (b) Inverse effective transport mass *m*_t_^–1^ of a given electron state vs its energy *E*. (c) Average inverse effective mass ⟨*m*_t_^–1^⟩_E_ versus thermal energy of hot carriers per electron Δ*E*. (d) Permittivity ε(ω) as a function of optical power density. Dashed line, approximate expression; solid line, exact.

The origin of intraband nonlinearities in TCOs arises from the nonparabolicity of the conduction band (CB) and the ensuing energy-dependent effective mass.^12,13,29^ To outline the effect, the dispersion of the nonparabolic effective mass can be approximated using **k.P** theory. In a material with bandgap *E*_g_ and effective mass m* at the bottom of the CB, one can normalize energy to *E*_g_/2 and to the wavevector  to obtain the dispersion law as shown in Figure 2a:
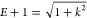
14or 2E + E^2^ = *k*^2^ which for unnormalized parameters becomes . Here, α = 1/*E*_g_ is a nonparabolicity parameter obtained under the assumption of two interacting bands. While actual experimental values of α vary, they are all close to the value of inverse energy gap for most TCO’s,^30^ hence (14) provides good estimate of the nonparabolicity.

The effective mass that is most relative to the optical properties is the transport mass, defined as
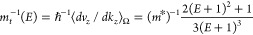
15where the averaging is done over a 4π solid angle, shown in Figure 2b. Similarly, the density of states is
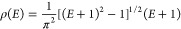
16

The density of carriers can be normalized to the low-temperature carrier density when the Fermi wavevector *k*_*F*_ = *k*_0_ or Fermi level at *E*_F0_ = √2 – 1.

The permittivity of the TCO is then described by the Drude formula:
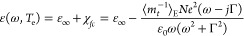
17where ε_∞_ is the permittivity due to bound electrons (playing the same role as *n̅*^2^ in the previous section), χ_fc_ is free carrier susceptibility, *N* is the free carrier density, and the average effective mass is
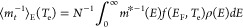
18where *f*(*E*_F_,*T*_e_) is the Fermi–Dirac distribution, *T*_e_ is electron temperature, and *E*_F_ is the temperature-dependent chemical potential. At low temperatures, the Fermi–Dirac function is step-like, and the value of the average effective mass can be found analytically as
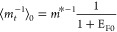
19

For heavily doped materials where the Fermi level resides in the linear portion of the CB (the case of most TCOs), *k*_F_ = *k*_0_ and *E*_F0_ = √2 – 1. Under this condition, we can define the low-temperature carrier density as *N*_0_ = *k*_0_^3^/3π^2^, and see that with *N* = *N*_0_, the average effective mass is ⟨*m*_*t*_^–1^⟩(0) = m^*-1^/√2. Now, neglecting Γ in eq 17 we obtain the expression for the frequency at which the real part of permittivity is zero as
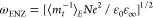
20

Under excitation, electrons at or below the Fermi level are transferred to higher energy states through free carrier absorption which then thermalize to heat the electron gas. Overall, the process results in an increased occupation of higher energy states that have a higher effective mass, see Figure 2b. Thus, the average effective mass of the electron gas tends to increase with excitation. In the limit of strong pumping, this would result in a decrease in the contribution of χ_fc_ in the Drude formula (see eq 17) until χ_fc_ ≈ 0 at the wavelength of operation, illustrating clear saturation of the modulation of permittivity. Thus, we can naively write that the permittivity of the material should follow a dependence similar to
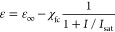
21

With some more work we can test this hypothesis.

To quantify the role of effective mass more specifically we determine the value of the average energy of an electron in the band as a function of electron temperature:
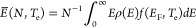
22

At low temperatures *E̅*(*N*_0_,0) = 0.26(*E*_g_/2) or about 0.5 eV for ITO. When heated, the energy of the average electron increases by
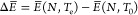
23where *T*_0_ is the lattice temperature. Combining eq 22 with eq 18, we can plot the dependence of the inverse effective mass on Δ*E̅*—the thermal energy per electron. This dependence is plotted in Figure 2c and can be linearized as
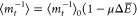
24

According to Figure 2c, the order of magnitude of slope μ approximately unity, or ∼1/*E*_g_ in absolute units. Given that the free carrier contribution to permittivity, as per eq 16, is proportional to ⟨*mt*^–1^⟩ it can be inferred that achieving a substantial change (e.g., 50%) in permittivity necessitates that each optically active electron (e.g., electrons within of the Fermi Level) acquire an energy on the scale of *E*_gap_, on the scale of 1 eV. This finding aligns with the proposition put forth in,^31^ suggesting that a significant alteration in the refractive index requires each optically active electron to acquire energy on the order of its binding energy, typically a few electron volts.

We can estimate this for ITO assuming an irradiance of 100 GW/cm^2^, a pulse width of 100 fs, *N* = ∼10^21^ cm^–3^ and a skin depth of 500 nm, as *W*_abs_ = *αIτ*_p_ = 0.2 kJ/cm^3^ producing an energy rise for each electron of *W*_abs_/*N* = 1.24 eV, which is approaching the region of saturation outlined by Alam et al.^9^ The energy density required to achieve this in TCO materials is thus on the scale of a KJ/cm^3^, which is relatively modest compared to other materials. The underlying reason is the relatively small effective mass of carriers in TCOs as well as our operation in the NIR rather than in the visible range.

Now, to determine the nonlinearity all we need is to establish the relation between the thermal energy per electron Δ*E̅* and the energy of the optical field per one electron, *U* = ε_0_ε_g_*E*_p_^2^/4*N* where *E*_p_ is the optical field in the TCO, and ε_*g*_ = ∂(*ωε*)/∂ω. Near the ENZ wavelength ε_*g*_ ≈ 2ε_*∞*_ ≫ ε, hence magnetic energy *U*_*M*_ = μ_0_*H*^2^/ 4*N* = ε_0_*εE*_p_^2^/4*N* (where μ_0_ is permeability) is quite small and can be neglected in this order-of-magnitude analysis. One can then write the rate equation for the thermal energy as
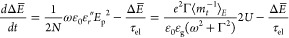
25Where eq 17 was used for the imaginary part of permittivity, and τ_el_ is the electron–lattice relaxation time. Using ε_g_ ≈ 2ε_∞_, we can write an approximate steady-state solution as
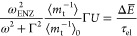
26

This equation relates Δ*E̅* and *U* since the inverse effective mass is a function of Δ*E̅* (Figure 2c). Using linearization via eq 24, we obtain
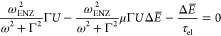
27

We can then introduce a saturation energy density per electron (once again in units of *E*_g_/2) as
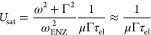
28which enables us to write the steady state solution as
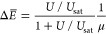
29

Combining with eq 17 can then write the real part of the permittivity as
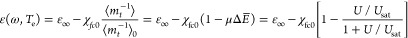
30where χ_fc0_ is free carrier susceptibility in the absence of heating. We can then define the value of the saturation power density as
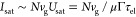
31where *v*_g_ is the group velocity, which then allows us to write
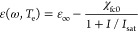
32which is in fact the same form that we hypothesized in eq 21.

For one of the most commonly studied TCO, indium-doped tin oxide (ITO), we can take E_*g*_ ∼ 3.8 eV, m* = 0.35*m*_0_,*k*_0_ = 3 nm^–1^, ε_∞_ = 3.9, *E*_F0_ = 0.78 eV, and carrier density *N* = 4.3 × 10^20^ cm^–3^.^32,33^ For low temperatures, this produces an ENZ frequency of 190 THz (λ_ENZ_ = 1570 nm); therefore, operating at around 1500 nm would place us in the transparent regime with ε_r_ = Re(ε) = ∼0.5. Furthermore, the term Γτ_el_ = τ_el_/τ_s_, where the momentum relaxation time τ_s_ is essentially the ratio of relaxation times T_1_/T_2_ that defines the enhancement factor of slow nonlinearities relative to the fast ones.^18^ For TCOs this ratio can be as high as 25 with τ_el_ = ∼50 fs and τ_s_ = ∼2 fs. Taking *v*_g_ = (1/4)*c*, and *μ = ∼*1/*E*_g_ the value of saturation power density can be then estimated as *I*_sat_ = ∼80 GW/cm^2^, which is a value consistent with prevalent experimental observations^14^ showing *I*_sat_ = ∼100 GW/cm^2^

Under these conditions, we illustrate the dependence of our estimated eq 21 versus intensity as shown in Figure 2d in the dashed lines. Clearly, this takes the form of a standard saturation curve. However, the curve for ⟨*m*_*t*_^–1^⟩ in Figure 2c deviates from the linear dependence assumed in eq 24, which is natural since an increase in effective mass cannot continue forever. Employing the first equality in eq 30 in conjunction with eq 26 yields a more precise result shown as in the blue solid line in Figure 2d. This result indicates a slightly delayed and lower saturation of permittivity. This observation stems from the inherent nonzero susceptibility of free carriers, regardless of their elevated thermal energy. A more comprehensive analysis would entail the consideration of electron transitions to higher bands within the TCO, leading to the observed “hard” saturation, as reported by Reshev and colleagues,^14^ but this is beyond the scope of this discussion.

A few points need addressing before concluding this section on effects in TCOs. First, despite the seemingly different origins of saturation in TCOs compared to the two-level system, a closer examination reveals a commonality. In both cases, the transfer of electrons to higher energy states leads to a decrease in cumulative oscillator strength. In the conventional saturation-type nonlinearity, the electrons in higher energy states exhibit negative oscillator strength since the transition originating from a higher energy state is downward. In TCOs, although the oscillator strength of electrons in higher energy states remains positive, it is reduced due to a larger effective mass. This is akin to the situation in multilevel atomic or molecular systems with excited-state absorption,^34^ where higher energy states still possess positive oscillator strength, albeit less than that of the ground state. Although in the case of the two-level system the electron population is strongly nonthermal while for TCOs we assume a thermal distribution for all times, the final result is similar. One should note that one can ascribe a separate temperature to the two levels in optically excited two level system and this temperature, different from the lattice temperature can be very high or even negative^35^ when population inversion is reached–thus playing the same role as electron temperature in TCOs.

Second, many experiments on TCOs utilized excitation with an ultrashort pulse of length τ_p_ < τ_el_. In such cases, one can estimate the response by replacing τ_el_ with τ_p_ in expressions eq 28 and eq 30. However, for exceedingly short pulses on the scale of the electric field cycle time, our present assumption of a thermal distribution of electrons in the conduction band may no longer be valid. In this case, the nonthermal distribution can give rise to localized population variations in *E*–*k* space that are described a response which is functionally similar to that of a two-level system.^36,37^ While beyond the scope of our focus here, such regimes are becoming of interest experimentally and theoretical descriptions must be handled appropriately.

Third, we remind readers that the discussion on TCOs and the ENZ regime is focused on the intrinsic nonlinearity of a bulk material at normal incidence, as this is the most fundamental response, and does not include any effects due to structural resonances or unique excitation such as Brewster or ENZ modes and boundary condition enhancement.^38^ One can take this response and add additional enhancement factors due to field confinement, slow light, resonance, etc. as has been shown in prior works. Although the material is strongly dispersive approaching the permittivity crossover point, giving rise to an apparent resonance effect in index commonly referred to as the Berreman mode, the description provided here remains valid in this regime. This is because the permittivity remains smooth and monotonic in the regime of the permittivity crossover, free from any apparent resonance, unlike the index, where modulation strength and sign will strongly depend upon the probe detuning from the permittivity crossover point. Thus, one can use the methods outlined here to determine the initial and final permittivity of the material, and subsequently index change, without requiring the introduction of resonance.

Lastly, we presume that the momentum scattering rate is constant throughout modulation. First, data on the dependence of the scattering rate on electron temperature in TCOs is not widely available. Unlike noble metals, where phonon-assisted scattering is the leading factor and gradually increases with electron temperature^39^ transport in TCOs is dominated by ionized donor scattering, which actually decreases with electron temperature.^40^ Moreover, for most TCOs a constant scattering rate is a good assumption because the scattering rate which is typically 1–5 × 10^14^ rad/s is much smaller than the plasma frequency which is typically 1–5 × 10^15^ rad/s. As a result, modifications upon the plasma frequency (e.g., N and ⟨*m*_*t*_^–1^⟩_E_) tend to dominate the permittivity response. However, we note that for more lossy materials, the temperature dependence of the scattering rate can become important, and lead to variations in sign, magnitude, and temporal response of the material, but in this order of magnitude analysis, we assume that changes in the scattering rate are small compared to changes in effective mass.

## Nonperturbative Regime in Ultrafast (Virtual) Nonlinearities

In the preceding sections, we have found that nonlinearities from real excitations (saturation-type, thermal, or photorefractive, etc.) can be analytically described using established models without resorting to perturbation theory. However, the challenge lies in addressing “virtual” or ultrafast^18^ nonlinearities, where light never gets absorbed, and, in fact does not lose its coherence.

Ultrafast nonlinear processes occur when detuning Δ*s*ignificantly exceeds broadening Γ. In third-order processes, they manifest as self- and cross-phase modulation, four-wave mixing, phase conjugation,^41^ and their variations. The bandwidth of these ultrafast processes is commensurate with , and can surpass hundreds of terahertz, albeit with a trade-off in magnitude. Experimentally, an ultrafast third-order nonlinearity is characterized by the nonlinear refractive index *n*_2_.^41,42^ This index, and χ^(3)^, can be either positive or negative. In solids, the nonlinear index is positive at lower energies (below half of the bandgap) and turns negative as the photon energy approaches the bandgap (or resonance for less dense media).^43−45^ Both positive and negative nonlinearities are typically treated perturbatively, and as mentioned in the introduction, what happens beyond perturbation theory is often shrouded in mystery. This fact appears indeed mysterious to us, as an exact nonperturbative analytical treatment of these nonlinearities has been readily available, ever since the introduction of dressed atom approach in 1960s by Cohen-Tannoudji and his co-workers.^46−49^

The dressed states approach, commonly applied to explain phenomena like the AC Stark effect, has traditionally concentrated on absorption and fluorescence spectra,^50^ neglecting changes in the refractive index. In this section, we demonstrate the application of the dressed state approach to derive exact nonperturbative expressions for ultrafast third-order nonlinearities, beginning with the negative nonlinearity associated with the proximity of photon energy to a resonant energy level or the bandgap energy in a solid.

### Negative Ultrafast Nonlinearity—“Virtual Saturation”

**Figure 3:**
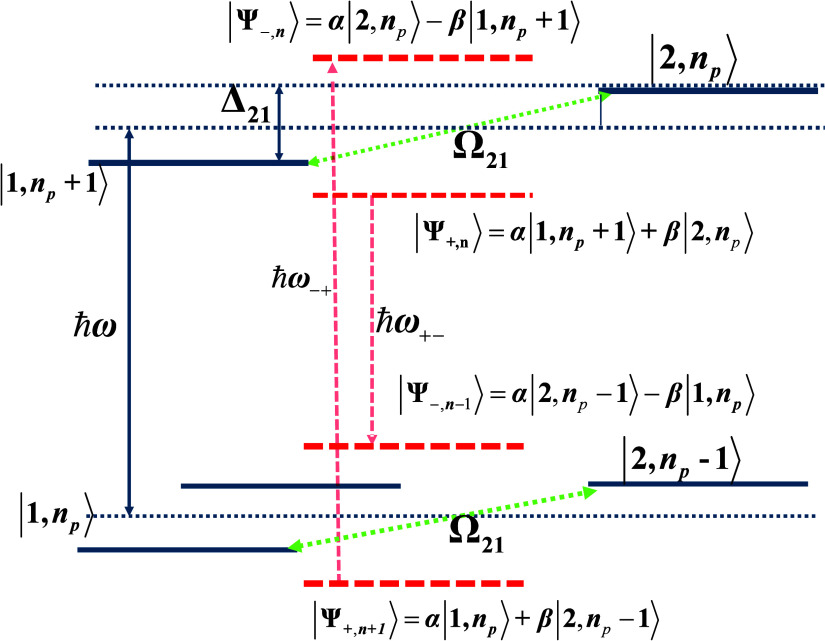
Negative ultrafast nonlinearity (“virtual saturation”). Coupled dressed states of the two-level system.

Consider a two-level system with states |1⟩ and |2⟩ separated by the energy in the presence of a strong pump field *E*_p_ cos ω*t* of frequency ω. The dressed states basis comprises the combinations of the matter and field states |1,*n*_p_⟩ and |2,*n*_p_⟩ containing *n*_p_ pump photons as shown in Figure 3. The two states |1,*n*_p_+1⟩ and |2,*n*_p_⟩ are coupled by the matter field interaction  where *d*_12_ is the aforementioned transition dipole matrix element, and Ω is the Rabi frequency. The Hamiltonian is then:
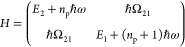
33

Diagonalization of this Hamiltonian matrix yields a series of dressed states:
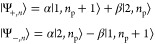
34where:
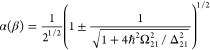
35and . The energies of the two states are
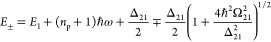
36

Since only transitions between two states with the same number of pump photons *n*_p_ are allowed, the strength of the spontaneous transition from the dressed state |Ψ_+,*n*_⟩ to the dressed state |Ψ_–,*n*-1_⟩ occurring at frequency can be found as |⟨1_ω+-_|⟨Ψ_+,*n*_|*H*_ep_|Ψ_–,*n*-1_⟩|0_ω+-_⟩|^2^, where *H*_ep_ is the electron–photon interaction Hamiltonian, and |1_ω+-_⟩ is the state with one spontaneously emitted photon of frequency ω_+-_. It thus involves the only allowed downward transition between |2,*n*_p_⟩ and |1,*n*_p_⟩ states. Therefore, this probability is proportional to the coefficient in form of β^4^. At the same time, the strength of the spontaneous transition between the states |Ψ_–,*n*_⟩ and |Ψ_+,*n*-1_⟩ is proportional to α^4^, occurring at frequency , i.e., much stronger. As a result, under the assumption that *n*_p_ is a very large number, the steady-state populations of the dressed states are
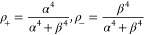
37and since α^2^ + β^2^ = 1 the population difference is
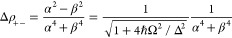
38

The absorption spectrum of the dressed system features a pronounced absorption line at frequency ω_–+_ > ω, characterized by an oscillator strength proportional to α^4^. Additionally, a weak gain line appears at frequency ω_+-_ < ω, with an oscillator strength proportional to β^4^. Notably, at frequency ω, both the absorption and gain lines contribute positively to the permittivity, allowing the following expression to be formulated:
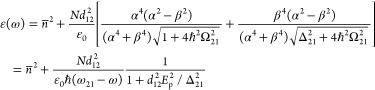
39

The expression eq 39 is equivalent to eq 5 when we introduce a different, “ultrafast” saturation intensity:
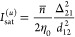
40

This process can be interpreted as the saturation of the index resulting from the existence of a “virtual population” density on the upper level:
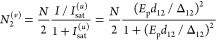
41The ultrafast nonlinearity attains significance and becomes “nonperturbative” when the pump field exceeds *E*_sat_ = Δ_21_/ d_12_. Notably, this threshold is essentially an atomic field scaled down by the resonance factor . In this context, the “nonperturbative” regime aligns with the well-described “strong coupling” regime found in the literature. Comparing eq 40 with eq 4 for the case of large detuning Δ ≫ Γ we obtain
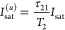
42

The ultrafast nonlinearity, usually much weaker than the slow nonlinearity by a factor of *T*_2_/ τ, shares an identical nonperturbative dependence as depicted in Figure 1b, albeit with a different (and typically much higher) saturation field. By expanding it into a series following eq 7, it results in odd-order nonlinearities akin to those in eq 8:
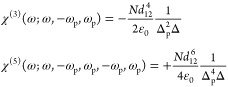
43

These expressions align precisely with what perturbation theory yields. However, as previously noted, utilizing them is unnecessary, given the ready availability of the exact solution in eq 39.

### Positive Ultrafast Nonlinearity—“Virtual Two-Photon Process”

**Figure 4:**
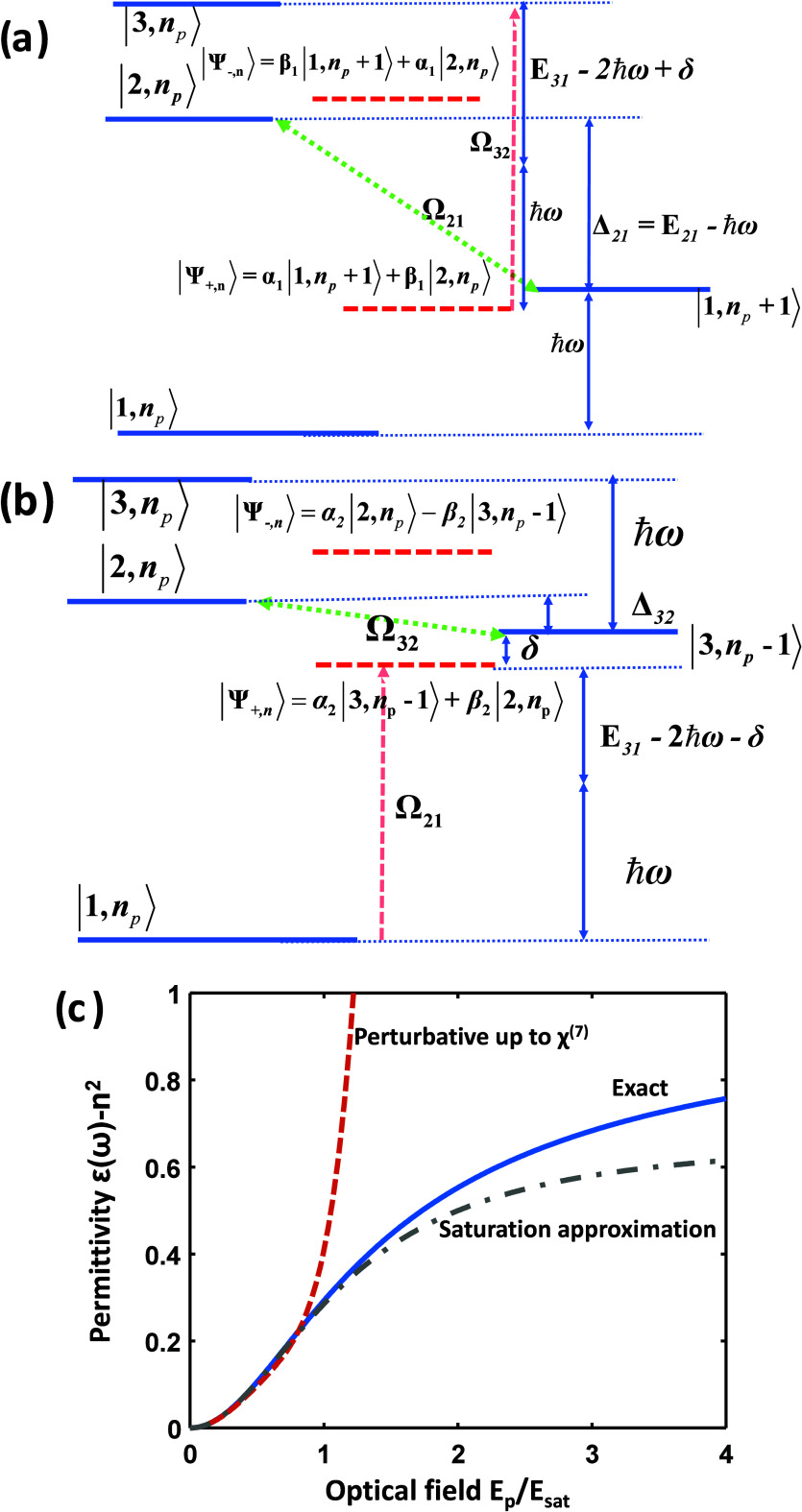
(a,b) Two contributions to ultrafast positive nonlinearity due to virtual two-photon processes. (c) The permittivity change as a function of the optical field. Solid line, exact result of eq 50; dashed line, perturbative approximation up to χ^(7)^; dot-dashed line, virtual saturation approximation of eq 33.

In practice, most practical nonlinear devices operate far from a single-photon resonance where the nonlinear index is positive and determined, as demonstrated in,^45,51,52^ by two-photon processes. This type of nonlinearity can also be precisely described using the dressed state approach. Consider a three-level system shown in Figure 4. Of the three levels, the only nonvanishing transition matrix elements are **d**_12_=*e*⟨1|**r**|2⟩ and **d**_23_=*e*⟨2|**r**|3⟩. Accordingly, in the presence of a strong pump one has a mixing of state |1,*n*_p_+1⟩ with state |2,*n*_p_⟩ as shown in Figure 4a, as well as mixing between states |2,*n*_p_ – 1⟩ and|3,*n*_p_⟩, as shown in Figure 4b.

The mixed state |Ψ_+,*n*_⟩ = α_1_|1,*n*_p_ + 1⟩ + β_1_|2,*n*_p_⟩ contains a contribution of the ground state defined as
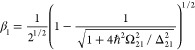
44where and , and its energy is
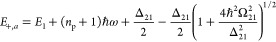
45

From this mixed dressed state, a transition to the upper state |3,*n*_p_⟩ occurs, and its strength is proportionate to β^2^, thereby yielding a nonlinear contribution to the susceptibility at the frequency ω:
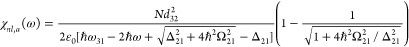
46

Turning now to Figure 4b, we note that the mixed state |Ψ_+,*n*_⟩ = α_2_|3,*n*_p_ – 1⟩ + β_2_|2,*n*_p_⟩ contains a contribution of the middle state |2⟩:
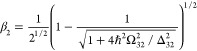
47where and , and the energy of this state is
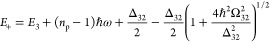
48

Now, a transition from the ground state |1,*n*_p_⟩ to |Ψ_+,*n*_⟩ enables a second contribution to the nonlinear susceptibility:
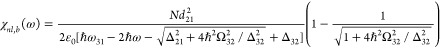
49

The formulas for nonlinear susceptibility eq 46 and eq 49 are precise; nonetheless, a simplification is feasible by assuming a substantial detuning from the two-photon resonance,is large, on the order of , as necessary to prevent two-photon absorption. Under these conditions, the overall nonlinear susceptibility becomes
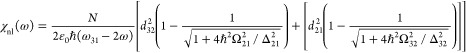
50

In Figure 4c, we plot the nonlinear contribution to susceptibility assuming for simplicity that one of the terms Ω_*ij*_/Δ_*ij*_ is much larger than the other, which is usually the case, while introducing the saturation field *E*_sat_ = ∼max(*d*_*ij*_/Δ_*ij*_). Clearly, the dependence on the field starts as quadratic but then saturates when the Rabi frequency exceeds the detuning–evidence of the ultrastrong coupling regime.^53^ Expanding this response into power series, one readily obtains nonlinear susceptibilities:
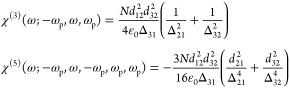
51

In Figure 4c, the perturbative solution up to the seventh-order susceptibility is plotted as the dashed line. In this case, the third-order susceptibility has a clear meaning and can be related to the ultrafast nonlinear index:
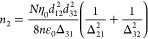
52

The physicality of higher orders may make sense if one is interested in odd harmonics, but beyond this case, one can perform a rearrangement of eq 50 and obtain a result for the intensity-dependent nonlinear index similar to
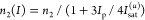
53where the ultrafast saturation intensity is defined by . As one can see from Figure 4c, the dot-dashed line, this approximation is almost exact for intensities as high as 3*I*_sat_ and saturates at the value only 25% less than the exact expression. Thus, it is sufficient to describe positive ultrafast nonlinearity as a saturation effect even beyond the perturbative regime.

It should be noted that in solids one deals with wide bands rather than with narrow lines, but still, the precise nonperturbative expressions for the ultrafast nonlinear response can be obtained by integrating eqs 39 and 50 over the entire Brillouin zone, as done in refs (45 and 52). Obviously, the onset of the nonperturbative regime in solids occurs at much higher pump intensities as detuning Δ becomes commensurate with the width of the band, rendering the resonant enhancement of nonlinearity ineffective. A partial restoration of this enhancement can be achieved through the utilization of quantum dots.^54^ However, a significant challenge arises due to the substantial inhomogeneous broadening present in quantum dots.

Furthermore, in solids, two-photon processes entail a combination of interband and intraband transitions within valence and conduction bands. The treatment in the momentum gauge involves the use of vector potential and momentum matrix elements *P*_*ij*_. However, a simplification can be achieved by substituting the relation between dipole matrix elements and momentum matrix elements *d*_*ij*_=*eP*_*ij*_/*mω* into relevant expressions. Additionally, employing the coordinate gauge for calculating nonlinearities, in quantum wells, considering a mixture of interband and intraband processes between confined states (as depicted in Figure 4, leads to results identical to those obtained for unconfined states in the momentum gauge as the width of quantum wells increases as shown in refs (55 and 56).

Note also that including fast oscillating instant intensity terms *I*(2ω) = ∼*E*_p_^2^*e* – 2^*iωt*^ into the denominator of eq 53 in addition to slow term *I*_p_ = ∼*E*_p_*E*_p_^*^ and using Taylor expansion would immediately yield permittivity changes proportional to all the even harmonics *e*^–2*miωt*^. When multiplied by the field, itself, these permittivity changes will yield all the odd harmonics as observed in ref (57).

Finally, as the pump intensity increases and one enters the nonperturbative regime for ultrafast nonlinearities, multiphoton absorption sets in and large “real” population of the excited states (bands) leads to saturation of refractive index according to expression similar to eq 5:
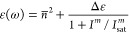
54where *m* is the order of muti-photon absorption, as experimentally observed in refs (51 and 58). Obviously, with the onset of absorption, the nonlinearity is no longer ultrafast. Hence, accessing the nonperturbative regime for ultrafast nonlinearities is most realistically feasible in atomic vapors.^59^ Additionally, leveraging pump field enhancement in diverse optical resonators can help achieve this regime.

However, a comprehensive discussion of all methods and approaches to achieve the nonperturbative regime in ultrafast nonlinearities exceeds the scope of this discourse. The first key point conveyed here is that a precise and physically transparent model for describing ultrafast nonlinearities, without relying on perturbation theory, has been available and can be utilized when necessary. The second key point is that the “nonperturbative” effect in ultrafast nonlinearity if effectively equivalent to ultra strong coupling which is a well-studied effect.

## Conclusions

In this paper, we’ve tackled how to handle changes in permittivity and refractive index when the standard perturbative methods fall short. The big problem with trying to expand a change in refractive index into a power series is that it predicts permittivity changes that are essentially infinite at high optical power densities. That is a no-go because it goes against what we know from observation—permittivity for materials in the optical spectrum typically stays between 1 and 10, even for very different materials.

If permittivity could change without limit, it would mean turning a material into something completely different under intense light. But that is just not realistic. As economist Herbert Stern pointed out,^60^ “If something cannot go on forever, it will stop.″ That applies here too—the change in permittivity will hit a limit eventually, regardless of the nonlinear mechanism at play, whether it is positive or negative, or how fast it is happening. Just like economic trends, there’s a cap on how much permittivity can change—it is bound to level off despite all the complexities involved.

We have considered both slow nonlinearities in which the incoming light gets absorbed, as well as ultrafast or instant nonlinearities in which absorption never takes place. While we have focused on odd-order effects, we note that the conclusions can be extended to other nonlinear processes, including even-order processes.

Our study reveals that the refractive index changes induced by all nonlinearities can be accurately characterized by a simple curve defined by two parameters: the saturation electric field (or irradiance) and the maximum change of permittivity at saturation. We have identified specific electric fields (or irradiances) at which saturation occurs for different types of nonlinearities. For ultrafast nonlinearities, saturation irradiances are determined solely by the transition dipole and detuning, while for slow nonlinearities, the characteristic lifetime of the excited state plays a crucial role. This lifetime can be associated with recombination time or electron–lattice relaxation time in the case of TCOs. Unlike previous approaches^14,16^ that relied solely on fitting experimental data without considering underlying physical processes, our model is grounded in well-defined material properties such as oscillator strength, characteristic times, and, for free carrier nonlinearities, the nonparabolicity of the bands. This provides a more robust and physically meaningful framework for understanding and predicting nonlinear optical phenomena in materials.

In addressing the question posed in the Introduction regarding the relevance of higher-order nonlinear susceptibilities in describing ultrastrong light–matter interactions, our findings indicate distinct scenarios for ultrafast and slow nonlinear phenomena.

For ultrafast phenomena, higher-order susceptibilities indeed hold significance as they can be linked to the hyperpolarizabilities of individual electronic states. This connection proves particularly valuable in contexts such as harmonic generation. However, it is worth noting that high odd harmonic generation can be achieved by incorporating time-dependent irradiance into the expression and subsequently expanding it in a Fourier series. A similar approach can be applied to even-order nonlinearities, resulting in the emergence of higher harmonics alongside saturation effects.

Conversely, in the case of slow nonlinearities, there exists no direct correlation between nonlinear coefficients and hyperpolarizabilities. In this context, higher-order nonlinearities lack physical significance beyond serving as Taylor series expansion coefficients and obscure the origins of the interactions at play. This is why many in the literature refer to these coefficients as “effective” coefficients, ultimately attempting to distinguish their difference from true ultrafast nonlinear susceptibilities and alert a reader that care must be taken when utilizing/interpreting the coefficient. Regardless, describing the index change using nonlinear index and saturation irradiance proves to be perfectly adequate for a wide variety of scenarios. We looked specifically at the case the TCOs in the ENZ regime and noted that that a conventional saturation curve can describe the nonlinearity to a good approximation. What is unique about TCOs at ENZ is that they are relatively easy to drive into the saturation regime. As outlined, *I*_sat_ = ∼100 GW/cm^2^ for many of the TCOs with ENZ in the NIR resulting in an energy density required for substantial permittivity change that is on the order of kJ/cm^3^. This is relatively modest compared to other materials^31^ and arises from the slow light enhancement, reasonably strong absorption, slow nonlinear response, and quite high damage threshold.^18^ Thus, TCOs do possess unique abilities to achieve strong permittivity and index modulation, as is well documented, but unique effects arising from nonperturbativeness are not among them.

To conclude, our comprehensive approach to nonlinear index change beyond the small perturbation limit is not intended to offer a simple solution for achieving significant index changes. Instead, we aim to provide valuable insights for researchers investigating strong light–matter interactions, with the ultimate objective of developing practical devices. By offering a unified treatment, we aspire to equip researchers with a deeper understanding of the underlying mechanisms governing nonlinear phenomena.
